# Evaluation of the effects of pre-exposure treatment with hydroxychloroquine on the risk of COVID-19 infection and on the efficacy of anti-COVID-19 vaccination during lupus or Gougerot-Sjögren’s disease: Prepcov multicentre trial

**DOI:** 10.1136/lupus-2024-001435

**Published:** 2025-03-05

**Authors:** Laurent Alric, Clara Brusq, Marion Migueres, Stephanie Faure, Pascal Lebray, Jean François Viallard, Dominique Chauveau, Laurent Sailler, Emilie Bérard, Grégory Pugnet, Patrice Cacoub, Prepcov study, Carle Paul

**Affiliations:** 1 Toulouse III University-Paul Sabatier, Toulouse, France; 2 Unité de Soutien Méthodologique à la Recherche (USMR), Service d’Epidémiologie Clinique et de Santé Publique, CHU de Toulouse, Toulouse III University-Paul Sabatier, Toulouse, France; 3 Virology Unit, CHU Toulouse, Toulouse, France; 4 Hepatogastroenterology, Montpellier University, Montpellier, France; 5 Hepatology Unit, Hopital Universitaire Pitie-Salpetriere, Paris, France; 6 Internal Medicine, Bordeaux III University, Bordeaux, France; 7 Kidney Disease Unit, Toulouse III University-Paul Sabatier Faculty of Health, Toulouse, France; 8 Purpan Hospital, Toulouse, France; 9 Service d’Epidémiologie et Santé Publique, Toulouse III University-Paul Sabatier Faculty of Health, Toulouse, France; 10 Internal Medicine Department, Toulouse III University-Paul Sabatier Faculty of Health, Toulouse, France; 11 Service de Médecine Interne et Immunologie Clinique, Hopital Pitie-Salpetriere, Paris, France

**Keywords:** COVID-19, Lupus Erythematosus, Systemic, Sjogren's Syndrome, Vaccination

## Abstract

**Objectives:**

Some patients with SLE or Gougerot-Sjögren’s disease (GSD) receive long-term treatment with hydroxychloroquine (HCQ), sometimes combined with immunosuppressive therapy (IS). This study sought to assess whether long-term HCQ therapy that had been initiated long before the COVID-19 pandemic had a protective or adverse effect on COVID-19 risk, severity of infection or immunity protection.

**Methods:**

This prospective multicentre study included 547 patients with SLE, GSD, autoimmune hepatitis, primary biliary cholangitis or cured viral hepatitis C divided into four groups according to HCQ (+/−) and IS (+/−) intake prior to the pandemic: HCQ+IS+ (n=112), HCQ+IS− (n=121), HCQ−IS+ (n=115) and HCQ−IS− (n=199). When COVID-19 vaccination was possible, patients were vaccinated as recommended. Vaccination efficacy was prospectively assessed on the basis of the postvaccination antibody titre.

**Results:**

Compared with HCQ+IS+ patients, HCQ−IS+ patients had a decreased risk of COVID-19 infection (p<0.001). Compared with HCQ+IS+ patients, HCQ−IS− patients had a decreased risk of contracting COVID-19 (p<0.001). Patients in the HCQ−IS+ or HCQ−IS− group had a lower risk of symptomatic or severe infection than HCQ+IS+ patients did (p=0.001 and p<0.001, respectively). Only patients who had two or more exposures (to vaccine and/or infection) had an increased likelihood of COVID-19 immunity after the last dose (p<0.001).

**Conclusions:**

HCQ treatment that was initiated before the pandemic did not protect against COVID-19 infection. Moreover, non-exposure to HCQ treatment (combined or not with IS) was associated with decreased risk of COVID-19 infection and of developing a symptomatic or severe infection. HCQ and IS do not influence the vaccine response. Only two or more doses of vaccine result in a good vaccine response.

**Trial registration number:**

NCT04481633.

**WHAT IS ALREADY KNOWN ON THIS TOPIC**

- A pre-exposure treatment strategy has been validated for use in the context of infectious diseases. Before the COVID-19 pandemic, some patients with SLE or Gougerot-Sjögren’s disease (GSD) received long-term treatment with hydroxychloroquine (HCQ). In the context of COVID-19 infection, a pre-emptive treatment strategy has never been tested prospectively. Despite its immunomodulatory properties, the influence of HCQ treatment on the anti-COVID-19 vaccine response is unknown.

**WHAT THIS STUDY ADDS**

- We demonstrated that pre-emptive HCQ treatment did not protect against COVID-19 infection.
- Non-exposure to HCQ treatment (combined or not with immunosuppressive therapy) decreased the risk of COVID-19 infection and the risk of developing severe disease.
- HCQ or immunosuppressive treatment did not modify vaccine-induced immunity.

**HOW THIS STUDY MIGHT AFFECT RESEARCH, PRACTICE OR POLICY**

- Our study suggests that, in the event of future SARS-CoV-type viral epidemics, HCQ should be used with caution, even given its validated indications for treating patients with SLE/GSD. Indeed, patients who receive long-term treatment with HCQ should be considered populations at independent risk of infection and therefore in need of appropriate protective measures.
- Patients receiving long-term HCQ treatment for a recognised indication can be reassured that this drug does not alter their vaccine response.

## Introduction

 In December 2019, starting in China before spreading worldwide, a pandemic linked to a new coronavirus responsible for coronavirus respiratory syndrome type II (SARS-CoV-2) emerged. The clinical spectrum of COVID-19 (as it was subsequently designated^1^) infection is very broad, encompassing asymptomatic forms as well as infections affecting the upper respiratory tract. While these are most often benign, some cases involve severe viral pneumonia, leading to death.^2^,^4^ More than half of humanity adopted containment measures to limit the spread of this virus. The pandemic caused a health crisis at a magnitude unseen for several centuries, bringing social relations and the economy to a standstill.^5^ People at high risk of complications and respiratory distress, such as those over 70 years of age or with pre-existing pathologies, were quickly identified.^1 3^ Immunocompromised patients are at greater risk of developing a severe form of infection. This pre-existing immunosuppression may be linked either to associated diseases or to treatments.^1 2^

In this critical situation, which was unprecedented both medically and socially, numerous treatments were proposed and tested, and the usual rules of clinical research were not always respected. Several non-specific antiviral treatments were tested but often performed poorly.^6^ Treatments using a combination of lopinavir and ritonavir with remdesivir exhibited clinical benefits.^7^ These are curative treatments for patients already infected with the virus, most of whom have severe forms of the disease or whose comorbidities put them at risk of developing a severe form of it.^8 9^

In severe cases, the second therapeutic target is to modulate overactivation of the immune system, which is partly responsible for the pulmonary alveolar lesions. Numerous studies have demonstrated an immune response to SARS-CoV-2 that exceeds regulatory capacity, with lesions probably immunoinduced, the mechanism of which remains poorly understood. Other strategies have therefore emerged to modulate this immune response, either through corticosteroid therapy or immunomodulatory biotherapy using drugs such as tocilizumab.^10^

At the frontier between antiviral and immunomodulatory activity, hydroxychloroquine (HCQ) has received particular attention. This led to much media hype about the efficacy of HCQ treatment in the context of COVID-19 infection.^11^,^13^ Preliminary studies, conducted in a hurried manner and using a questionable methodology, seemed to show the efficacy of HCQ in treating COVID-19 infection. Subsequent studies, which were conducted on a sound scientific basis, did not confirm this beneficial effect, and also revealed severe side effects in some cases.^14^,^16^ However, HCQ has been widely used for several decades in the treatment of autoimmune diseases and inflammatory rheumatism, with an immunomodulatory effect. This drug is not considered an immunosuppressant (IS).^17^

Long before the COVID-19 pandemic, some patients^18 19^ with SLE or Gougerot-Sjögren’s disease (GSD) received long-term treatment (sometimes for a number of years) with HCQ, which has marketing authorisation for this type of autoimmune disease. In certain situations, patients with SLE/GSD may be treated with HCQ in combination with an IS. In such circumstances, the risk of immunodepression and susceptibility to infection is higher.^20^ Other autoimmune diseases, such as autoimmune hepatitis (AIH), are not treated with HCQ, but patients may or may not receive immunosuppressive therapy.^21^ Finally, patients with cured viral hepatitis C or primary biliary cholangitis (PBC) are not treated with HCQ or immunosuppressive therapy. In the absence of significant hepatic fibrosis, these patients have a status close to that of the general population.^22 23^ The present population of patients with SLE and/or GSD, therefore, includes individuals who had long been exposed to HCQ under its regulatory approval and well before the COVID-19 pandemic, and also includes natural control groups not exposed to this drug.

Pre-exposure treatment has been validated in the context of a number of infectious diseases. For example, the combination of tenofovir and dipivoxil along with emtricitabine has been shown to significantly reduce the rate of HIV infection in at-risk populations.^24^ In the context of COVID-19 infection, a pre-emptive treatment strategy has never been tested prospectively, either in the general population or in the targeted immunosuppressed population, where numerous studies have shown that, regardless of the causative pathology, COVID-19 infection is more severe. In the general population, but also, more specifically, in patients receiving immunosuppressive therapies, prophylactic pharmacological treatment would be particularly useful. Only two studies^25 26^ conducted on national databases reported that long-term treatment with HCQ had no effect on the mortality of COVID-19 infection in patients with rheumatoid arthritis or SLE. HCQ has been shown not to be a curative treatment for COVID-19 infection. A strategy of pre-exposure treatment with HCQ, however, has never been tested prospectively. In addition, despite its immunomodulatory properties, the influence of HCQ treatment on the anti-COVID-19 vaccine response is unknown.

The aim of this prospective multicentre study was to verify whether treatment with HCQ in a cohort of patients with SLE/SGJ, in their usual regulatory marketing authorisation, taken over the long term well before the onset of the pandemic, could reduce the risk of COVID-19 infection, the intensity of the disease or the anti-COVID-19 vaccine response.

## Patients and methods

**Figure 1:**
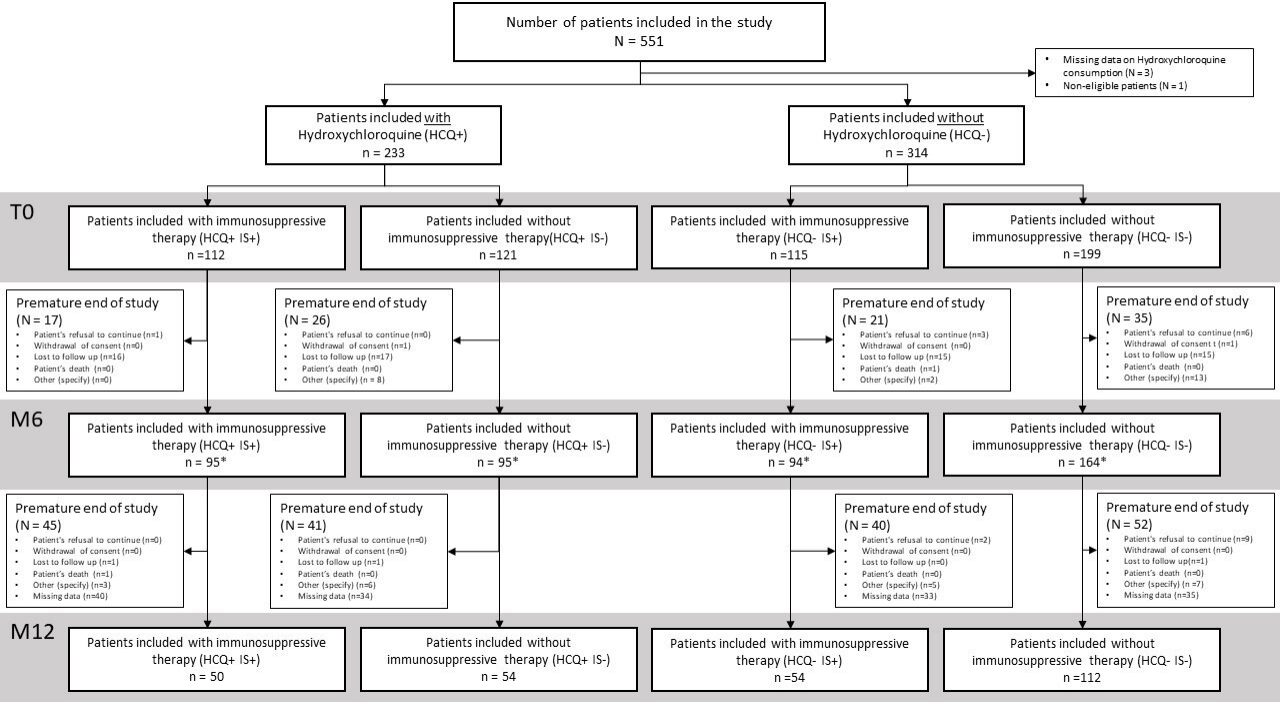
Prepcov flow diagram for study inclusion. Some patients did not have a visit at M6*, but did have a visit at M12, and were not considered as study exits at M6. HCQ+, hydroxychloroquine treatment; HCQ−, without hydroxychloroquine treatment; IS+, immunosuppressive treatment; IS−, without immunosuppressive treatment; M6, 6-month follow-up visit; M12, 12-month follow-up visit; T0, study inclusion visit.

The Prepcov study (ClinicalTrials.gov NCT04481633) is a multicentre study with prospective follow-up that includes patients who were treated between September 2020 and December 2023 at one of 12 centres across France in Toulouse, Bordeaux, Montpellier and Paris. This study compared a group exposed to HCQ who had begun treatment before the COVID-19 pandemic and continued treatment during the pandemic with a group of patients not taking HCQ for more than 12 months (figure 1). The effects of HCQ on COVID-19 infection and vaccine response were evaluated.

The first group consisted of patients with SLE or GSD who were regularly taking HCQ 400 mg/day well before and during the pandemic, with or without IS such as azathioprine, methotrexate, mycophenolate mofetil (MMF) or cortisone. The control group consisted of patients not taking HCQ. This group included patients seen as part of the usual follow-up of PBC or who were cured of hepatitis C virus (HCV) without significant liver fibrosis. Patients with IS with AIH whose usual treatment was azathioprine and patients with SLE or SGJ receiving azathioprine, methotrexate or cortisone but not HCQ for any reason were also included. The diagnosis of SLE or GSD according to the American College of Rheumatology criteria^18 19^ or AIH or PBC was established according to international criteria.^22 23^ Patients were seen every 6 months for the duration of the study, with a questionnaire to determine whether COVID-19 infection had occurred, with an assessment of its severity, and serology to determine the presence of anti-COVID-19 antibodies (Abs) and the Ab level when serology was positive. When COVID-19 vaccination was possible, patients were vaccinated as recommended with Comirnaty (Pfizer-BioNTech) or Spikevax (Moderna).

Prior to the vaccination period, COVID-19 infection was identified either by positive nasopharyngeal PCR or by the appearance of anti-spike Abs on two successive serologies. During the period of generalised vaccination, COVID-19 infection was identified on the basis of positive PCR results or positive serology with anti-spike and anti-SARS-CoV-2 nucleocapsid protein Ab tests, indicating contact with the virus. In terms of vaccine protection, a postvaccination Ab level of >140 Binding Antibody Unit (BAU)/mL was used.

The symptomatic or severe nature of COVID-19 infection was assessed by the physician using a questionnaire completed at each visit, which was based on criteria such as the duration of symptoms, intensity of clinical manifestations, use of oxygen therapy and need for hospitalisation.

All patients over 18 years of age provided written consent to participate in the study.

### Inclusion criteria

This multicentre study with a prospective follow-up of 12 months included 547 patients divided into four groups according to whether they were taking HCQ with (+) or without (−) IS, such as cortisone, azathioprine, methotrexate or MMF (figure 1).

*Patients in the HCQ group(n=233)* were those with SLE or GSD receiving HCQ therapy for more than 3 months prior to the COVID-19 outbreak, that is, since at least December 2019, and continuing it for the duration of the study. They were divided into two subgroups:

- Group HCQ+IS−, n=121: patients were taking HCQ but not taking IS.
- Group HCQ+IS+, n=112: patients were taking IS in combination with HCQ.

*In the non-HQC group (n=314)*, patients who were not receiving HCQ were divided into two subgroups:

- Group HCQ-IS−, n=199: no HCQ or IS taken for more than 12 months, HCV cured for more than 12 months or PBC with normal liver blood tests. Non-significant hepatic fibrosis assessed either by liver biopsy or transient elastography with non-significant hepatic fibrosis, Metavir score <F2.

- Group HCQ−IS+, n=115: patients with SLE or GSD or AIH not taking HCQ but treated with IS for more than 3 months prior to the COVID-19 outbreak.

### Exclusion criteria

- The use of IS such as anti-CD20 Abs or cyclophosphamide in the 6 months prior to inclusion, refusal of a blood test for anti-COVID-19 Abs, pregnancy or breastfeeding, plasma or recent blood transfusion and lack of health insurance coverage.

### Main objective

- To evaluate whether long-term use of HCQ that began before the COVID-19 pandemic had a protective effect against COVID-19 infection in patients with SLE or GSD.
- To determine whether an IS such as azathioprine, methotrexate, MMF or cortisone has adverse effects in terms of the risk of SARS-CoV-2 infection and the risk of developing symptomatic or severe disease.

### Secondary objectives

- The postvaccination Ab titre was evaluated to determine if it was at a protective level stratified by the treatments received (HCQ and/or IS). The threshold of 140 BAU/mL is considered^27^ to be the threshold above which vaccine protection is effective.

### Virological methods

#### SARS-CoV-2 molecular assays

During the study period, COVID-19 diagnosis was widely and freely available to everyone through nucleic acid amplification tests (NAATs) via nasopharyngeal sampling. Laboratory-based NAATs target at least two different viral sequences.^27^ SARS-CoV-2 molecular detection in nasopharyngeal samples was performed using the Aptima SARS-CoV-2 transcription-mediated amplification assay (Hologic, San Diego, California) or a laboratory-developed test based on real-time PCR with the Panther Fusion module (Hologic).

#### SARS-CoV-2 serological assays

##### Abs against the SARS-CoV-2 spike protein

We used the Wantaï SARS-CoV-2 Ab ELISA (Beijing Wantaï Biological Pharmacy Enterprise, Beijing, China) or the Alinity SARS-CoV-2 IgG II Quant (Abbott Ireland, Diagnostics Division, Sligo, Ireland) assay to quantify Abs directed against the SARS-CoV-2 spike antigen. For each method, samples above the upper limit of quantification were diluted with the appropriate dilutant, and the result was calculated. The raw Antibody Unit (AU)/mL data were converted into BAU/mL units as previously described.^27^

##### Abs against the SARS-CoV-2 nucleocapsid protein

Anti-nucleocapsid (anti-N) Abs were detected using the Alinity SARS-CoV-2 immunoglobulin G (IgG) assay (Alinity, Abbott). An IgG index ≥1.4 indicates a positive serological result for anti-N Abs.

### Statistical analysis

Before doing any analysis, we checked the power of the study. Considering that we had observed 103 patients with COVID-19 infection during the 12-month follow-up, we could analyse up to 10 risk factors of COVID-19 infection, which was quite satisfactory.

First, a descriptive analysis was conducted according to the four groups (HCQ+IS+, HCQ+IS−, HCQ−IS+, HCQ−IS−). Qualitative variables are presented as frequencies and percentages. Quantitative variables are presented as the means and SDs. No imputation methods were implemented to replace missing data. The analysis of different judgement criteria followed the same methodology. A descriptive analysis of potential confounding factors was performed by groups (exposed vs non-exposed; ie, HCQ+ vs HCQ−) to verify the balance of the groups. The potential confounding factors tested included age, known diabetes, obesity (body mass index (BMI) >30 kg/m²), corticosteroid use, polymedication (≥2 treatments, excluding those for SLE, GSD, AIH, HCV or PBC) at inclusion and number of doses (vaccine and/or infection) for vaccine immunity judgement criteria. A bivariate analysis of potential confounding factors in relation to the judgement criterion was also implemented. For this purpose, we conducted comparison tests of percentages (χ^2^ test or Fisher’s exact test, depending on theoretical counts) for qualitative variables and comparison tests of means (Student’s t-test if normality and variance equality assumptions were met) or distributions (Mann-Whitney U test for non-parametric data) for quantitative variables.

Variables significantly associated with the primary judgement criterion in bivariate analysis (at a threshold of 0.20) and with exposure (HCQ+ vs HCQ− groups) were introduced into a logistic regression model, as were the four study groups (HCQ+IS+, HCQ+IS−, HCQ−IS+, HCQ−IS−). A stepwise backward selection method was then used to obtain the final model. Nested intermediate models were compared using the likelihood ratio test and the Akaike information criterion. Interactions between the independent variables of the final model and the four study groups were sought in the final model. None were significant. The goodness of fit of the model to the data was also verified using the Hosmer-Lemeshow test. The significance threshold was p<0.05. The analyses were performed using Stata software (Statistical Software: Release 18.0, Stata, College Station, Texas, USA).

## Results

A 12-month prospective follow-up from September 2020 to December 2023 included patients at 12 centres across France (table 1). Descriptive analysis by groups (exposed/non-exposed; ie, HCQ+/HCQ−) was carried out to verify the group balance. Factors of imbalance were considered potential confounders. The demographic and epidemiological characteristics of the different groups were not significantly different, which hampered the statistical analysis (table 1). However, patients in the HCQ−IS− control group were significantly older and consumed more alcohol than did those in the HCQ group (p<0.001). Other differences were linked to the initial pathology, with, for example, a predominance of women expected in the SLE/GSD group (p<0.001). The median duration of HCQ use in patients with SLE/GSD at inclusion was 72 months (IQR: 36–141) and the median evolution time was 120 months (55–216). The median dose of cortisone at inclusion was the same in HCQ+IS+ (5 mg/day (5.00–9.50)) and HCQ−IS+ (5 mg/day (5.00–11.25)) patients. Identically, the median dose of methotrexate at inclusion was the same in HCQ+IS+ and HCQ−IS+ patients (15 mg/week (10–15)). The median dose of azathioprine at inclusion was higher in HCQ+IS+ (100 mg/day (75–125)) than in HCQ−IS+ (75 mg/day (50–100)) patients. The median duration of cortisone use in HCQ+IS+ and HCQ−IS+ patients at inclusion was 36 months (7–120) and 30 months (12–84), respectively. The median duration of methotrexate use in HCQ+IS+ and HCQ−IS+ patients was 21 months (6–30) and 31 months (14–60), respectively. The median duration of azathioprine use in HCQ+IS+ and HCQ−IS+ patients was 43 months (31–120) and 74 months (46–111), respectively.

**Table 1:** Demographic and clinical characteristics of the patients

	HCQ+IS+(n=112)	HCQ+IS−(n=121)	HCQ−IS+(n=115)	HCQ−IS−(n=199)	P value
Age, years (mean, SD)	47.84 (13.226)	45.73 (13.605)	55.52 (30.474)	61.47 (12.135)	<0.001
Female sex	104 (92.9%)	107 (88.4%)	90 (78.3%)	117 (58.8%)	<0.001
Metropolitan France origin	77 (71.3%)	101 (84.9%)	97 (85.8%)	160 (82.1%)	0.021
BCG vaccination	89 (84.8%)	102 (89.5%)	95 (88.0%)	180 (91.4%)	0.364
Influenza vaccination	48 (44.0%)	45 (38.1%)	67 (58.3%)	73 (36.7%)	0.002
BMI, kg/m² (mean, SD)	24.85 (5.999)	24.50 (4.849)	25.50 (5.451)	25.09 (4.072)	0.234
Known diabetes	6 (5.4%)	1 (0.8%)	8 (7.0%)	14 (7.0%)	0.084
Chronic respiratory disease	6 (5.4%)	3 (2.5%)	8 (7.0%)	8 (4.0%)	0.379
High blood pressure	26 (23.4%)	12 (9.9%)	23 (20.2%)	52 (26.1%)	0.005
Chronic renal failure	13 (11.7%)	3 (2.5%)	4 (3.5%)	14 (7.0%)	0.016
Chronic heart disease	10 (9.0%)	2 (1.7%)	5 (4.4%)	10 (5.0%)	0.080
Neurological disease	14 (12.6%)	15 (12.4%)	11 (9.6%)	14 (7.0%)	0.308
Cancer history	8 (7.2%)	3 (2.5%)	5 (4.4%)	9 (4.5%)	0.394
Current smoker	21 (19.1%)	20 (16.5%)	15 (13.0%)	39 (19.6%)	0.481
Alcohol consumption	2 (1.8%)	9 (7.4%)	8 (7.0%)	32 (16.1%)	<0.001
Other comorbidity	34 (30.9%)	32 (26.4%)	27 (23.9%)	62 (31.2%)	0.489
Cortisone therapy	69 (61.6%)	0 (0.0%)	48 (41.7%)	0 (0.0%)	<0.001
Polymedicated patient	72 (64.3%)	49 (40.5%)	60 (52.2%)	71 (35.7%)	<0.001
Vaccine and/or infection					
0	31 (27.7%)	38 (31.4%)	29 (25.2%)	50 (25.1%)	NA
1	16 (14.3%)	5 (4.1%)	6 (5.2%)	14 (7.0%)	NA
2	23 (20.5%)	36 (29.8%)	30 (26.1%)	69 (34.7%)	NA
3	24 (21.4%)	28 (23.1%)	41 (35.7%)	55 (27.6%)	NA
4	13 (11.6%)	14 (11.6%)	8 (7.0%)	11 (5.5%)	NA
5	5 (4.5%)	0 (0.0%)	1 (0.9%)	0 (0.0%)	NA
2 doses or more					
Vaccine or infection	65 (58.0%)	78 (64.5%)	80 (69.6%)	135 (67.8%)	0.247

BMIbody mass indexHCQ+, hydroxychloroquine treatment; HCQ−, without hydroxychloroquine treatment; IS+, immunosuppressive treatment; IS−, without immunosuppressive treatmentNA, not applicable

### Primary objective: COVID-19 infection risk and disease severity as a function of treatments received

In patients with SLE or GSD, we assessed whether long-term use of HCQ that began before the COVID-19 pandemic had a protective effect against COVID-19 infection and developing a symptomatic or severe form of the infection. We also determined whether an IS such as azathioprine, methotrexate, MMF or cortisone had an adverse effect on HCQ-induced protection in terms of the risk of infection and the risk of developing a symptomatic or severe form.

**Figure 2:**
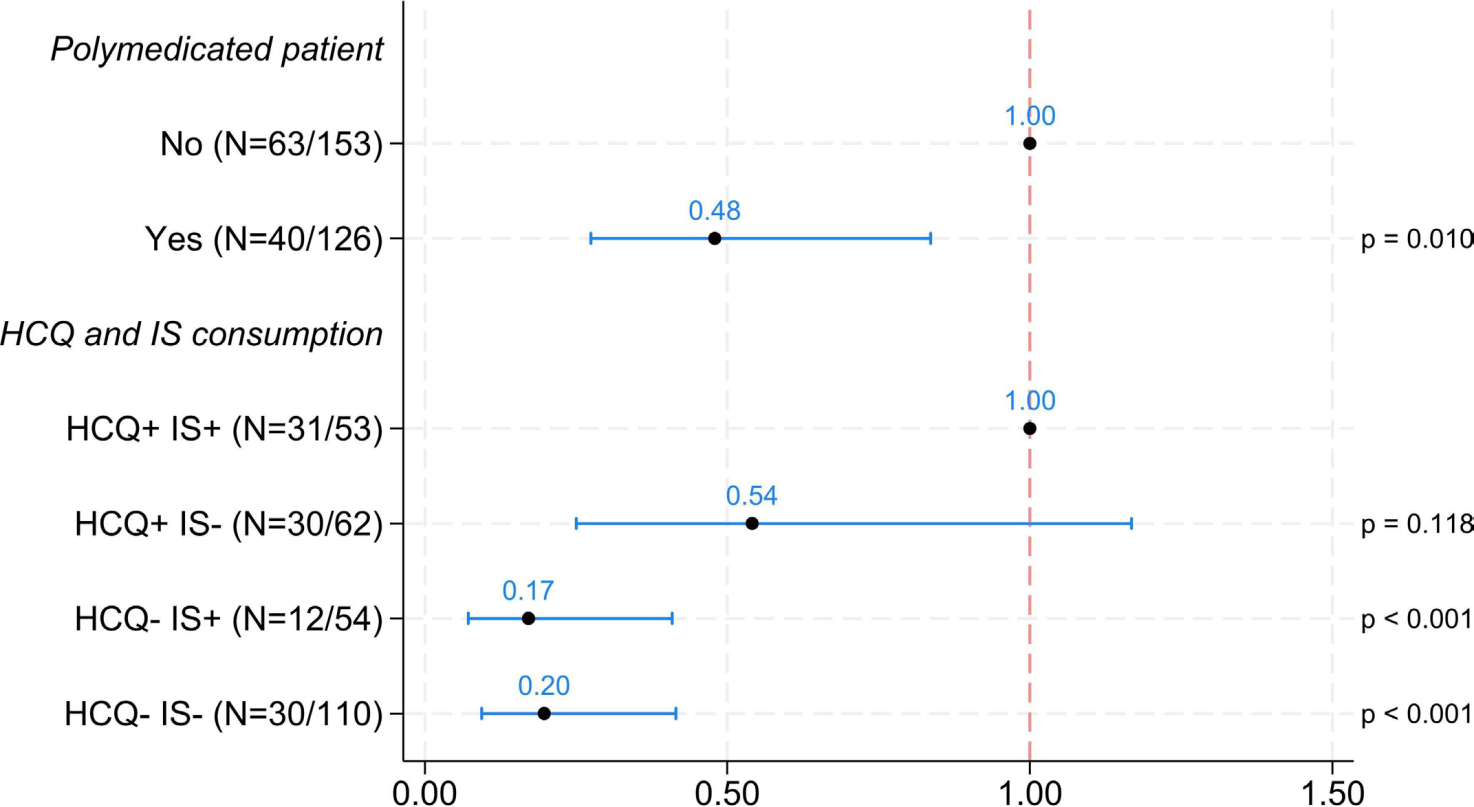
Risk of COVID-19 infection depending on the treatments received. ORs and logistic regression models for serology or PCR results in favour of COVID-19 infection. Categories are presented as N=number of events, number of patients. The risk of having serology or PCR results indicating COVID-19 infection in the different groups was analysed. The initial model included the variables: patient aged over 65, cortisone consumption, HCQ and IS consumption, polymedicated patient. HCQ+, hydroxychloroquine treatment; HCQ−, without hydroxychloroquine treatment; IS+, immunosuppressive treatment; IS−, without immunosuppressive treatment.

In the first analysis, the risk that serology or PCR indicated COVID-19 infection in the different groups was analysed (figure 2). Compared with non-polymedicated patients, polymedicated patients had a 52% lower risk (multiplied by 0.48, OR=0.48, 95% CI 0.27; 0.84) of having a serology or PCR in favour of a COVID-19 infection (figure 2). This reduction in risk was statistically significant (p=0.010). Patients in the HCQ−IS+ group had an 83% lower risk (multiplied by 0.17, OR=0.17, 95% CI 0.07; 0.41) of having serology or PCR in favour of COVID-19 infection than HCQ+IS+ patients did (figure 2). This reduction in risk was statistically significant (p<0.001). Patients in the HCQ−IS− group had an 80% reduced risk (multiplied by 0.20, OR=0.20, 95% CI 0.09; 0.42) of having serology or PCR in favour of COVID-19 infection compared with HCQ+IS+ patients (figure 2). This reduction in risk was statistically significant (p<0.001).

**Figure 3:**
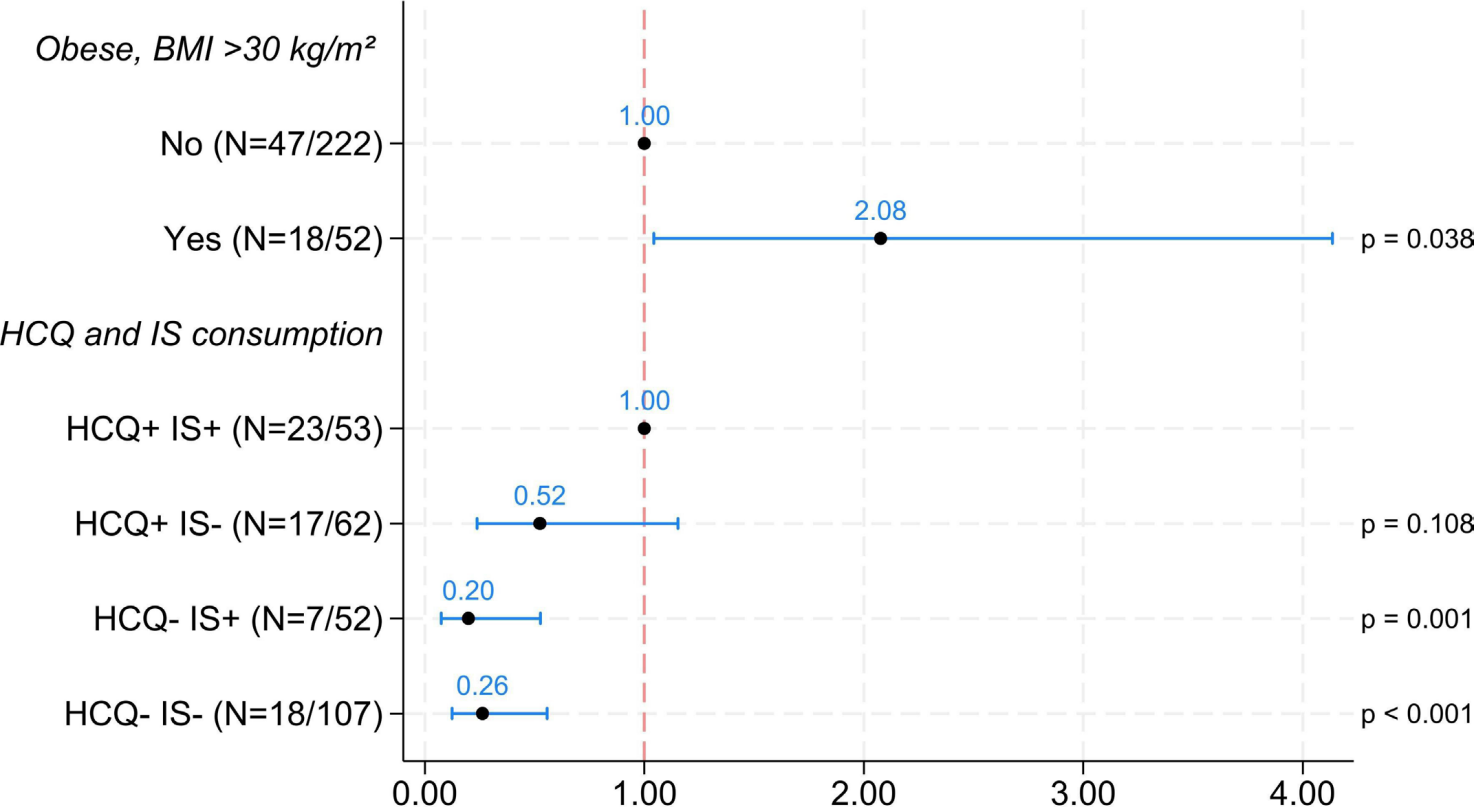
Severity of COVID-19 infection depending on the treatments received. ORs and logistic regression model for symptomatic or severe forms of COVID-19 infection during the study. Categories are presented as N=number of events, number of patients. The initial model included the variables: patient aged over 65, cortisone consumption, HCQ and IS consumption, obesity (body mass index (BMI) >30 kg/m^2^). The symptomatic or severe nature of COVID-19 infection was assessed by the physician using a questionnaire completed at each visit. HCQ+, hydroxychloroquine treatment; HCQ−, without hydroxychloroquine treatment; IS+, immunosuppressive treatment; IS−, without immunosuppressive treatment.

We then analysed the risk of developing a symptomatic or severe form of infection according to the treatments received via questionnaires completed at each medical visit (figure 3). Patients with obesity had a 2.08-fold increased risk (OR=2.08, 95% CI 1.04; 4.14) of developing a symptomatic or severe form of COVID-19 during the study compared with patients without obesity (figure 3). This increased risk was statistically significant (p=0.038). Patients in the HCQ−IS+ group had an 80% lower risk (multiplied by 0.20, OR=0.20, 95% CI 0.08; 0.53) of having a symptomatic or severe form of COVID-19 during the study than HCQ+IS+ patients did. This reduction in risk was statistically significant (p=0.001). Patients in the HCQ−IS− group had a 74% lower risk (multiplied by 0.26, OR=0.26, 95% CI 0.12; 0.56) of having a symptomatic or severe form of COVID-19 during the study than HCQ+IS+ patients did (figure 3). This reduction in risk was statistically significant (p<0.001).

### Secondary objective: vaccine protection according to the treatments received

**Figure 4:**
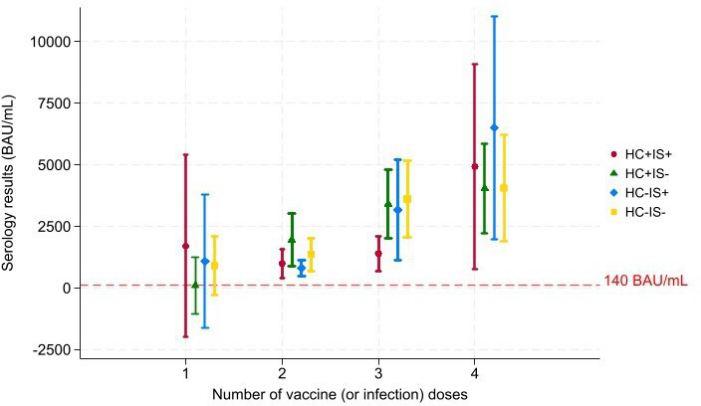
Secondary objective: vaccine protection according to the treatments received. ORs and logistic regression model for vaccine protection after the last dose. The threshold of 140 BAU/mL is considered to be the threshold above which vaccine protection is effective. The initial model included the variables: HCQ and IS consumption and two doses or more (vaccine and infection) during the study. The graph shows the mean serology values and their associated 95% CIs; the dotted red line represents 140 BAU/mL. BAU, Binding Antibody Unit; HCQ+, hydroxychloroquine treatment; HCQ−, without hydroxychloroquine treatment; IS+, immunosuppressive treatment; IS−, without immunosuppressive treatment.

We assessed the proportion of patients with effective postvaccination immunity according to the treatment received, which had been started before the pandemic (figure 4). Our measurements were based on postvaccination Ab levels in the different groups (Ab rate >140 BAU/mL). There was a proportional increase in Ab levels with increasing number of vaccine doses in all groups. Adherence to vaccination was good in all groups (between 68% and 75%).

HCQ and IS consumption was not significantly associated with having effective vaccine immunity (>140 BAU/mL) after the last dose (figure 4). Patients who received two or more doses (vaccine and infection) during the study had an 8.8-fold increased risk (OR=8.80, 95% CI 3.92; 19.72) of having effective vaccine immunity (>140 BAU/mL) at the end of the last dose compared with patients who received fewer than two doses (figure 4). This increased risk was statistically significant (p<0.001).

## Discussion

Our study revealed that HCQ treatments initiated long before the pandemic and prescribed in line with HCQ’s usual marketing authorisation in patients with SLE/GSD did not protect against COVID-19 infection but had no influence on the vaccine response.

A pre-exposure treatment strategy has been validated for use in the context of a number of infectious diseases. In the case of COVID-19 infection, a pre-emptive treatment strategy has never been tested prospectively in either general or targeted populations. Several empirical curative treatments for COVID-19 infection, such as ivermectin, metformin or HCQ, have been used in emergency situations at the peak of the epidemic.^8^ None of these treatments were effective. Other treatments have since been used on a more solid scientific basis.^7^,^10^ A distinction is made between antiviral therapeutic strategies and those aimed at countering the pulmonary immune storm induced by viral infection.^10^

To the best of our knowledge, only two studies^25 26^ have shown the absence of an impact of pre-emptive HCQ treatment on the risk of infection or mortality from COVID-19 infection. The first study, carried out in England^25^ on a national database, is interesting but has the weakness of this type of study, which the authors emphasise. Unlike our prospective study, in that national, population-based study,^25^ there were many potential limitations, such as a lack of certainty about the classification of the disease, the reality of treatment uptake or even the lack of certainty about COVID-19 infection. In the second study,^26^ it was also observed that patients with rheumatic diseases in Brazil taking HCQ had the same risk of COVID-19 infection as those not treated with HCQ. This study also has several limitations, as it was carried out with a telephone, and the patients were not seen during a medical consultation. In that study, there was no virological confirmation of COVID-19 infection, a critical point that may have influenced the results.^26^ The control group was composed of cohabitants who may have had non-homogeneous measures of social distancing. The follow-up period was also short.^26^ In our study, many of these potential pitfalls observed in studies using databases were eliminated, as our cohort was prospective, with patients physically seen during a medical consultation, with a long medical follow-up and a perfectly documented medical record. The strengths of our multicentre prospective study with control groups on a more limited sample are the precise knowledge of the clinical profile of the patients included, the nature of the drugs taken, the very early start date of HCQ treatment and the certainty of infection by COVID-19 and its consequences, taking into account comorbidities. It therefore seems that our study is complementary to these two database studies.^25 26^

In 2019/2020, there was a scientific rationale for testing HCQ in the treatment of COVID-19 infection, but this was undertaken in a way that departed from the usual scientific rules for clinical testing. The subsequent media frenzy was just one example of the excesses both scientists and ordinary citizens witnessed during this troubled period, when humanity felt threatened.^5 28 29^ HCQ has long been used against an infectious disease (malaria), as an immunomodulator in SLE and/or GSD and in other rheumatological diseases. Under all of these conditions, with doses of 400 mg/day, its safety is acceptable.^17 20^ The effect of HCQ on the immune system is linked to the inhibition of lysosomal activity in dendritic cells and B lymphocytes by modifying antigen presentation and modulating the production of interleukin 1 (IL-1), IL-6 and tumour necrosis factor-alpha. In addition to this immunomodulatory effect, HCQ can have a direct antiviral effect in vitro by modifying membrane fusion receptors, preventing the cellular penetration of coronaviruses while interfering with the glycosylation of angiotensin-converting enzyme, a cellular receptor for COVID-19. Similarly, some studies have suggested that HCQ blocks virus integration into endosomes by modifying the cellular pH and activating lysosomal proteases, leading to virus degradation.^30 31^ Given that HCQ’s properties are at the frontier of anti-infectious and immunomodulatory activity, it seems legitimate to put an end to this controversy by prospectively and rigorously evaluating the pre-exposure efficacy of HCQ against COVID-19 infection and its effects on the anti-COVID-19 vaccine response. An assessment of these effects on populations exposed and unexposed to this drug and their control groups with or without immunosuppressive treatment presented itself as a good strategy for doing so.

Our choice of populations for this study was therefore pragmatic. We had a population of patients with SLE and/or GSD on long-term treatment well before the start of the COVID-19 pandemic, and some of them had been on HCQ for many years in the recognised treatment indications for SLE/GSD. Some of these patients with SLE/GSD may have been treated with HCQ in combination with immunosuppressive therapy. Other autoimmune diseases, such as AIH, are not treated with HCQ, but patients may receive immunosuppressive therapy. Finally, patients with cured HCV or PBC are neither treated with HCQ nor given immunosuppressive therapy, and in the absence of significant hepatic fibrosis, these patients have a status close to that of the general population.^22 23^ We chose patients, rather than healthy subjects such as blood donors, as the group not exposed to HCQ. Indeed, the period in which populations were ordered to socially distance and remain at home is a variable to be taken into account when judging the beneficial effect of pre-exposure treatment or adherence to vaccination. In the case of healthy subjects, such as blood donors, who by their nature are younger subjects free from disease, it is likely that adherence to confinement or vaccination could be more variable than in the case of subjects who knew they were ill, such as those with a disease.

In this study, we demonstrated the unfavourable effect of pre-emptive HCQ treatment on COVID-19 infection. Indeed, patients taking immunosuppressive drugs alone had an 83% lower risk of COVID-19 infection than patients taking immunosuppressive drugs and HCQ did. Patients taking neither HCQ nor immunosuppressive drugs had an 80% lower risk of COVID-19 infection than patients taking HCQ and immunosuppressive drugs did. Moreover, the same results were observed for the severity of COVID-19 infection, since patients receiving immunosuppressive therapy without HCQ had an 80% reduced risk of having a symptomatic or severe form compared with patients receiving HCQ and IS. Patients taking neither HCQ nor IS had a 74% lower risk of developing symptomatic or severe COVID-19 disease than patients taking HCQ and IS did. We therefore showed that long-term HCQ taken before the onset of the pandemic did not protect against occurrence or severity of COVID-19; instead, HCQ had a deleterious effect on both of these. In contrast, in our study, immunosuppressive therapy did not increase the risk or severity of COVID-19 infection. According to other studies, pre-existing immunosuppression is a factor in the severity of COVID-19 infection, as is obesity, which is a risk factor for intensive care unit admission.^1 2^ In all of these studies, immunosuppression was associated with anticancer chemotherapy, heavy immunosuppressive therapy or severe comorbidities such as cancer^2^ or morbid obesity. The patients in our cohort received less severe immunosuppressive treatments, mostly monotherapy, with little corticosteroid therapy and no severe obesity (ie, BMI <40 kg/m²).

The main bias in our study was related to the absence of double-blind randomisation. To compensate for potential confounding biases in the comparison of HCQ-exposed and non-exposed patients, we performed multivariate logistic regression analyses adjusted for potential confounding factors. Despite the accumulation of scientific data against the use of HCQ in the treatment of COVID-19, the most unyielding defenders of this treatment have argued that its failure was linked to the introduction of HCQ treatment too late after contamination by COVID-19. This argument is demonstrably false. The strength of our study lies in the fact that we included patients treated with HCQ for a recognised medical indication, often for several years well before the pandemic and with good compliance, since we had HCQ blood tests for many patients as part of their SLE or GSD routine follow-up (data not shown). Our study suggests that, in the event of future SARS-CoV-type viral epidemics, HCQ should be used with caution, even given its validated indications for treating patients with SLE/GSD. Indeed, our study shows that these patients who receive long-term treatment with HCQ should be considered populations at increased risk of infection and therefore require appropriate protective measures.

Containment measures, barrier measures and large-scale vaccination have helped to break the epidemic peaks of contamination.^32^,^34^ In our study, adherence to vaccination was good, since the majority of patients in all groups accepted vaccination. We also observed that HCQ or immunosuppressive treatment did not affect vaccine immunity. This immunity is influenced only by the number of doses of vaccine received. Notably, some patients were also infected with COVID-19 during the course of the study, which may have increased the vaccine response. In fact, our study revealed that patients who received two or more doses of the vaccine and/or had an infection had an 8.8-fold improvement in effective vaccine immunity compared with patients who received fewer than two doses of the vaccine. In our study, the presence of Abs directed against COVID-19 appeared to be the most reliable criterion for confirming viral infection by COVID-19, particularly when the Abs are directed against the nucleocapsid of the virus. We also considered that an Ab threshold above 140 BAU/mL was associated with protective immunity.^27^ Nasopharyngeal PCR has been shown to be a low-sensitivity test with false negatives.^8 35^ Conversely, serological tests appear to have good sensitivity and specificity. The kinetics of the appearance and disappearance of viral RNA detected by PCR in nasopharyngeal secretions shows that viral RNA detection is transient and that the serological profile is much more stable over time for determining whether a patient has been infected with COVID-19. Several studies have shown that Abs appear less than a month after viral infection and appear stable for several weeks or even months thereafter.^8 35^ We have chosen this criterion as a criterion of certainty for the diagnosis of past infection with COVID-19, and our study points to a proportional rise in the level of Abs against COVID-19 as a function of the number of vaccine doses, without any influence from immunosuppressive treatment. However, patients receiving long-term HCQ treatment for a recognised indication can be reassured that this drug does not alter their vaccine response.

*In summary*, HCQ treatment initiated before the pandemic did not protect against COVID-19 infection. Non-exposure to HCQ treatment (combined or not with immunosuppressive therapy) decreased the risk of COVID-19 infection and of developing severe disease. Furthermore, HCQ did not significantly influence the vaccine response. Receiving two doses of vaccine (and/or having had an infection) resulted in a good vaccine response, including for patients on immunosuppressive therapy.

## Data availability statement

All data relevant to the study are included in the article or uploaded as supplementary information.
